# A Cohort Study on Self-Reported Respiratory Symptoms of Toner-Handling Workers: Cross-Sectional and Longitudinal Analysis from 2003 to 2008

**DOI:** 10.1155/2014/826757

**Published:** 2014-02-27

**Authors:** Hiroko Kitamura, Niina Terunuma, Shizuka Kurosaki, Koichi Hata, Masashi Masuda, Takeshi Kochi, Nobuaki Yanagi, Tadashi Murase, Akira Ogami, Toshiaki Higashi

**Affiliations:** ^1^Department of Work Systems and Health, Institute of Industrial Ecological Sciences, University of Occupational and Environmental Health, 1-1 Iseigaoka Yahatanishi-ward, Kitakyushu 807-8555, Japan; ^2^Department of Haematology, Tokai University School of Medicine, Japan

## Abstract

This study examines the relationship between toner-handling work and its health effects on self-reported respiratory symptoms. The subjects were 1,504 male workers in a Japanese toner and photocopier manufacturing company. Personal exposure measurement, pulmonary function tests, chest X-ray examination, measurement of biomarkers, and a questionnaire about self-reported respiratory symptoms were performed annually. This study discusses the questionnaire results. We found that the toner-handling group showed significantly higher prevalence of breathlessness than the never-toner-handling group. The significant reduction of pulmonary function and fibrosis change in the chest X-ray examination associated with breathlessness were not observed. However the morbidity of asthma was higher compared to the Japanese population in both of the toner-handling group and the never-toner handling group, the effect of toner exposure was not clarified. Nevertheless, while the toner exposure levels in the current well-controlled working environment may be sufficiently low to prevent adverse health effects, further studies are needed to assess the more long-term latent health effects of toner exposure.

## 1. Introduction

Toner is an ink powder used in laser printers and photocopiers to form printed text and images on paper. According to the safety data sheet for the commonly used black toner that was issued by manufacture, the main components of the toners are polyester resin, carbon black, iron oxide, and a few other elements. In the 1990s, progress in digital technology led personalization of laser printers and multifunctionalization of photocopiers, and, after this, printers and photocopiers have become essential things in our modern lives. As a result, the amount of toner consumption also increased significantly. In this situation, a case of siderosilicosis which occurred after 6 years of exposure to toner dust from photocopier machine was reported in 1994 in *The Lancet* [1]. Thereafter, the case of granulomatous pneumonitis and mediastinal lymphadenopathy which occurred after 18 months of work in a newspaper agency was reported in 1996 [2, 3]. Armbruster et al. concluded that photocopier toner dust caused chronic lung disease. In 1996, carbon black, a toner component, was reevaluated as Group 2B that is possibly carcinogenic to human, based on experiments on rats by the International Agency for Research on Cancer (IARC) [4]. According to Szozda [5], the association between exposure to carbon black and pneumoconiosis was showed in the 1950s based on a few cases of simple pneumoconiosis. Szozda [5] reviewed the health studies in work forces occupationally exposed to carbon black and concluded that pneumoconiosis was considered as an occupational disease of workers exposure to carbon black in its production and usage. However, the information in these studies was insufficient to verify the actual risk of carbon black and toner on humans because of the fact that most articles were case reports of specific diseases, the acute effects of uncontrolled exposure conditions, and animal experiments with higher exposure concentrations than would be encountered in the workplace. As the past articles only presented cross-sectional studies and in vitro or in vivo experiments, the scientific evidence to evaluate the magnitude of chronic lung effects from toner exposure was insufficient. A longitudinal well-designed study on humans was needed; we started a 10-year cohort study to investigate the relationship between toner exposure and its health effects. We set these respiratory diseases as endpoints: lung fibrosis, pneumoconiosis, chronic obstructive pulmonary disease (COPD), and lung cancer. This paper is the result of a 5-year follow-up from 2003 to 2008. The aim of this study is to examine the relationship between toner-handling work and its health effects on self-reported respiratory symptoms based on the results of the first half of this 10-year cohort study.

## 2. Materials and Methods

### 2.1. Study Design

The present study was designed to investigate long latency respiratory diseases, that is, pneumoconiosis, COPD, lung cancer, and so forth, related to toner exposure through a 10-year follow-up period. In addition to the annual health check, the following 5 examinations were also conducted once a year: (1) personal exposure measurement, (2) questionnaire of self-reported respiratory symptoms, (3) chest X-ray examination, (4) blood analysis and urinalysis, and (5) pulmonary function test. In particular, we would examine self-reported respiratory symptoms in this paper.

### 2.2. Subjects

The subjects were 1,504 male workers under 50 on April 1, 2003, in a Japanese toner and photocopier manufacturing company. The subjects consisted of toner-handling group and never-toner-handling group as the referents. The toner-handling group included workers who engaged in 5 work categories: toner development, toner manufacturing, photocopier development, toner and photocopier recycling, and customer service. The never-toner-handling group only included workers who have not engaged in toner-handling works, since they entered in the company. Most of the never-toner-handling workers were engaged in desk work in the offices. Several workers were transferred from the never-toner-handling group to the toner-handling group during the survey period.

### 2.3. Measurement of Toner Exposure Level

The annual plan of personal exposure measurements was made by the health supervisor every year. About 5 workers in each workplace were randomly selected and personal exposure measurements were conducted based on the Working Environment Measurement Standards [6]. In Japan, the definition of respirable dust in this standard was revised from equal to or less than 7 *μ*m to 4.5 *μ*m in 2004. Along with this revision, the personal dust sampler was changed from 2005, which was when the 3rd survey was conducted. A filter holder for a personal total and respirable dust sampler, Model PS-43, Roken type (Shibata Scientific Technology, Ltd.) (flow rate 1.5 L/min) with glass fiber filters, PTFE binding, T60A20 type *φ*25 mm (Tokyo Dylec Corp.), was used until 2004, and a filter holder for a personal dust sampler, Model NWPS-254 (Shibata Scientific Technology, Ltd.) (flow rate 2.5 L/min), with glass fiber filters, PTFE binding, T60A20 type *φ*25 mm (Tokyo Dylec Corp.), has been used since 2005. The detector for the personal dust sampler was placed in the workers' breathing zone. If the worker used personal protective equipment (PPE), measurement was performed outside the PPE. Respirable dust trapped in the filter was measured with an electric balance, and the measured quantity was divided by the total aspirated air volume to calculate the respirable dust concentration. In order to compare with the threshold limit value (TLV) as defined by the American Conference of Governmental Industrial Hygienists (ACGIH), which is based on an allowable exposure averaged over a normal 8-hour workday or 40-hour workweek, an 8-hour time weighted average (TWA8 h) was calculated by the formula indicated by the ACGIH [7].

### 2.4. Questionnaire about Self-Reported Respiratory Symptoms

In Japan, the Japanese version of ATS-DLD-78 Adult Questionnaire [8] was created by the Environment Agency. We use this Japanese version questionnaire and added some questions, that is, questions about a work history of toner-handling work, handling of organic compounds in this year, and a medical history in this year and administered. The following 5 chronic symptoms were assessed in this study because these chronic symptoms were related to respiratory diseases that we set as endpoints: persistent cough, persistent phlegm, persistent cough and phlegm, wheezing without asthmatic response, and breathlessness.

### 2.5. Statistical Analysis Method

Subjects defected work history data were excluded from the analysis. The exposure measurement results were presented as a geometric mean (GM) with geometric standard deviation (GSD) and median with quartile 1 and quartile 3 because the data exhibited a log-normal distribution. In the cross-sectional study, the chi-square test was used to compare the prevalence of symptoms. In the longitudinal analysis, we performed logistic regression analysis using generalized estimating equations (GEE), which was the statistical model for repeated measures data analysis to examine the association between chronic respiratory symptoms and toner exposure. Dependent variables were self-reported respiratory symptoms, and independent variables were toner exposure, smoking status, handling of organic compounds, morbidity of asthma, morbidity of allergic rhinitis, morbidity of allergic dermatitis, age, and pulmonary function indices, which were percent predicted vital capacity (%VC) and percent predicted forced expiratory volume in one second (%FEV_1.0_). Using the prediction formula for Japanese adult males described by the Japanese respiratory society, which is VC(l) = 0.045 ∗ height (cm) − 0.023 ∗ age − 2.258 and FEV_1.0_(l) = 0.036 ∗ height (cm) − 0.028 ∗ age − 1.178, we calculated %VC and %FEV_1.0_ [9]. *P* < 0.05 was regarded as statistically significant. All analyses were performed in SPSS20.0J for Windows (SPSS Inc.).

## 3. Results

### 3.1. Subjects

Among 1,504 subjects, toner-handling group was 1071 workers and never-toner-handling group was 415 workers at the start of the study. 18 subjects were excluded from the analysis due to lack of the work history data. We followed up 1,328 workers over 5 years and the follow-up rate was 89.4%. When participants withdrew from the study, we obtained the reasons in writing and confirmed that no subjects withdrew due to the specific respiratory diseases that we set as endpoints. During the study period, 5 subjects in the toner-handling group and 1 subject in the never-toner-handling group died of cerebrovascular attack, ischemic heart disease, and other diseases. We did not find a significant difference in mortality between the toner-handling group and the never-toner-handling group (chi-square test *P* value = 0.54).

### 3.2. Measurement of Toner Exposure Level

The results of personal exposure measurement stratified by toner-handling work categories were provided in Table 1. Although the GM of TWA8 h varied widely from 0.018 mg/m^3^ in photocopier development to 0.569 mg/m^3^ in toner and photocopier recycling in the 1st survey, the differences among the toner-handling work categories almost disappeared as the survey proceeded. Even the highest GM, the value was well below the acceptable value of 3.0 mg/m^3^ which was recommended by the ACGIH as the TLV-TWA of particles not otherwise specified.

### 3.3. Characteristics of the Subjects

The characteristics of the subjects in the 1st survey are shown in Table 2. The age distribution of all subjects was from 19 to 50 years and the mean age was 38.6 years old. The toner-handling group was 1.2 years older than the never-toner-handling group. The smoking rate was 50.7% and 45.8% in the toner-handling group and the never-toner-handling group, but statistically significant differences were not observed (chi-square test *P* value = 0.22). There were no significant differences in morbidity of asthma, allergic rhinitis, and allergic dermatitis between the toner-handling and the never-toner-handling groups. The toner-handling group tended to use organic compounds more often than the never-toner-handling group. Workers who engaged in customer service used an alcoholic organic solvent to clean photocopier machines. Though workers who engaged in toner manufacturing responded that they used organic compounds during toner production process, organic compounds were used in the sealed production line and workers were not exposed in steady work.

### 3.4. Prevalence of Self-Reported Respiratory Symptoms

#### 3.4.1. Cross-Sectional Study

The prevalence of self-reported respiratory symptoms in the toner-handling group and the never-toner-handling group is shown in Table 3. Age was a factor which affected the subjective symptoms, especially breathlessness; as the study proceeded, the difference of the mean age between the 2 groups became small: 1.09 years in the 1st survey (Student's *t*-test *P* value < 0.01), 0.72 years in the 2nd survey (*P* value = 0.06), 0.63 years in the 3rd survey (*P* value = 0.10), 0.56 years in the 4th survey (*P* value = 0.15), 0.49 years in the 5th survey (*P* value = 0.21), and 0.52 years in the 6th survey (*P* value = 0.19). Considering the results of a cross-sectional study, the difference of age might not become a major factor. The prevalence of persistent cough, persistent phlegm, and persistent cough and phlegm did not show any significant differences between the tone-handling group and the never-toner-handling group through the study period. However, wheezing without asthmatic response in the 2nd survey and breathlessness in all survey showed statistically obvious differences between the 2 groups.

#### 3.4.2. Longitudinal Study

Firstly, we conducted GEE using self-reported respiratory symptoms as dependent variables and toner exposure, smoking status, handling of organic compounds, morbidity of asthma, morbidity of allergic rhinitis, morbidity of allergic dermatitis, and age as independent variables. Toner exposure showed significant effect on breathlessness increasing (odds ratio 1.87). Current smoking showed strong effects on all symptoms increasing: persistent cough (odds ratio 2.42), persistent phlegm (odds ratio 3.08), persistent cough and phlegm (odds ratio 3.31), wheezing without asthmatic response (odds ratio 2.73), and breathlessness (odds ratio 3.16). Subjects with asthma and allergic rhinitis showed a high possibility to complain of symptoms. Aging significantly affected persistent phlegm, wheezing without asthmatic response, and breathlessness increasing. The GEE results are shown in Table 4.

Secondly, we conducted GEE using self-reported respiratory symptoms as dependent variables and toner exposure, %VC, and %FEV_1.0_. The results are shown in Table 5. In this analysis, toner exposure showed significant effect to increase breathlessness (odds ratio 1.98). %VC was used as the indicative of restrictive disorder and %FEV_1.0_ was obstructive disorder. %VC and %FEV_1.0_ showed the opposite effects. As the %VC increased 1%, the prevalence of persistent cough and phlegm increased 1.04 times and the prevalence of wheezing without asthmatic response increased 1.08 times. As the %FEV_1.0_ increased 1%, the prevalence of persistent cough and phlegm decreased 0.95 times and the prevalence of wheezing without asthmatic response 0.92 times.

### 3.5. Incidence of COPD, Pneumoconiosis, and Lung Cancer

On the basis of the self-reported medical history collected by the questionnaire, there were 14 COPD subjects, no pneumoconiosis subjects, and no lung cancer subjects at the beginning of the study, and 14 COPD subjects were excluded from the analysis. There were 9 subjects who developed COPD, no subjects who developed pneumoconiosis, and 1 subject who developed lung cancer during the study period. The incidence rate of COPD in all subjects was about 10.7/10,000(9/8407) person per year and the incidence rate of lung cancer in all subjects was 1.2/10,000(1/8506) person per year. Based on the toner exposure category of the end of the follow-up year, the incidence rate of each group was calculated. The incidence rate of COPD of the toner-handling group was 8.1/10,000(5/6189) person per year and that of the never-toner-handling group was 18.9/10,000(4/2113) person per year. The incidence rates of lung cancer was 1.6/10,000(1/6251) person per year in the toner-handling group. The incidence rate ratio of COPD in the toner-handling group and in the never-toner-handling group was 0.43. The result was different from the assumption at the beginning of this study.

## 4. Discussion

We found that the toner-handling group was significantly more like to report breathlessness. Breathlessness was a typical symptom of COPD and pneumoconiosis and the severest symptom in this study. The relation of inhalation of chemicals to COPD and inhalation of fine particles to pneumoconiosis was known well. Toner is a particulate chemical of about 6 to 8 microns in size, so a possibility that inhalation of toner would cause COPD and pneumoconiosis could not be denied. In the subjects who complained breathlessness, the relationship between toner exposure and airflow limitation that is, %FEV_1.0_ less than 80%, was considered. The results were shown in Table 6. The consisted tendency between breathlessness and %FEV_1.0_ was not observed and the significant difference due to toner exposure was also not observed by qui-square test. There were no subjects who had classified chest X-ray into category over 1/1 according to the ILO international classification of radiographs of pneumoconiosis [10] through the study period. However, it was hard to consider that organic changes such as COPD and pneumoconiosis had developed from the results of the clinical examination, it is necessary to follow the symptoms in the future study.

According to a comprehensive survey of living conditions which was a national survey conducted by Ministry of Health, Labour and Welfare [11], the prevalence of breathlessness in Japanese males aged 20 to 59 was about 2.9%; breathlessness was defined as the subjective breathlessness for the past few days in this national survey. Although the definition of the symptom was differed from our study, the prevalence of breathlessness in our subjects was higher. There was a possibility of information bias [12]. Subjects might have become sensitive to their respiratory symptoms, especially severer one, since we explained the aim of this cohort study at the time of informed consent.

According to Fukutomi et al., morbidity of current asthma in Japanese males was 5.5% in 20s, 4.9% in 30s, 4.3% in 40s, and 2.3% in 50s [13]. Morbidity of asthma in our study was 9.8% in all subjects and 10.0% and 9.4% in the toner-handling group and the never-toner-handling group, respectively. Our subjects showed about twice morbidity. There was one case report which described allergic asthma induced by xerographic toner [14]. However, the actual mechanism of asthmatic reaction to toner is still unclear. We consider that the differences arose by discrepancy of the definition of asthma. In our study, questionnaire was a self-administer type and diseases were assumed to be occurred by self-assessment. Fukutomi et al. defined current asthma patients as those who diagnosed by a physician and shown an active symptom in the last 12 months. Unfortunately, we cannot reestimate using the same definition again. What we can do is to observe the morbidity of asthma carefully in the future.

As the reasons for withdrawing from the study, being busy with work, oversea transfers, the unpleasantness of pulmonary function tests, and other issues were raised. The subjects who felt unpleasantness from pulmonary function tests might be in not good lung condition. Most of the respiratory diseases set as endpoints require a long time to develop to a level diagnosed clinically. It is a limitation of this study that subjects were relatively young and further information about those who withdrew from the study could not be obtained.

Toner exposure level in this study was well below the acceptable value of 3.0 mg/m^3^ recommended by the ACGIH [7] as the TLV-TWA of particles not otherwise specified. After reviewing the results of the 1st survey, the company and workers took measures to reduce toner exposure. The measures included modifying tools and equipment, better safety rule enforcement, and changes in handling materials and work procedures. Workers who engaged in toner manufacturing and toner and photocopier recycling were also instructed to ensure using dust protective masks and replacing the filters over a period determined by safety rules in the company. Certainly, the actual exposure was lower than outside the dust protective mask; from the point of the safety and health, we measured toner dust concentration outside the dust protective masks. There was a possibility of the overestimation of risk of toner exposure. In order to make a suitable judgment, we would continue this cohort study.

Toner-handling works tended to have relatively heavy physical work load than nonhandling works, so workers with serious health problems might have retired or been reassigned to different works. We had to take the healthy worker bias [15] into consideration, and our classification that is, the toner-handling group and the never-toner-handling group, seemed to make sense in the point of reducing the influence of this bias.

## 5. Conclusion

We found that the toner-handling group showed significantly higher prevalence of breathlessness than the never-toner-handling group. The significant reduction of pulmonary function and fibrosis change in the chest X-ray examination associated with breathlessness were not observed. However the morbidity of asthma was higher compared to the Japanese population in both of the toner-handling group and the never-toner handling group, the effect of toner exposure was not clarified. There are no previous reports of a longitudinal epidemiological study focusing on toner exposure. This study presents the first half of a 10-year cohort study that started in 2003. Further studies are needed to assess the more long-term latent health effects of toner exposure.

## Figures and Tables

**Table 1:** Personal exposure measurement.

Toner-handling work category	1st survey	2nd survey	3rd survey	4th survey	5th survey	6th survey
Toner development						
Sampling case^#^	19	17	11	11	5	7
GM^##^ (mg/m^3^)	0.026	0.026	0.014	0.012	0.015	0.011
(GSD^##^)	(1.974)	(2.059)	(1.507)	(1.355)	(1.069)	(1.299)
Median (mg/m^3^)	0.018	0.020	0.014	0.013	0.015	0.011
(quartile 1, quartile 3)	(0.015, 0.039)	(0.015, 0.041)	(0.010, 0.016)	(0.011, 0.015)	(0.014, 0.015)	(0.009, 0.016)
Toner manufacturing						
Sampling case	61	65	72	60	74	71
GM (mg/m^3^)	0.075	0.064	0.036	0.043	0.028	0.025
(GSD)	(3.446)	(3.742)	(2.963)	(3.447)	(2.847)	(2.909)
Median (mg/m^3^)	0.045	0.044	0.035	0.023	0.018	0.016
(quartile 1, quartile 3)	(0.032, 0.159)	(0.021, 0.156)	(0.014, 0.065)	(0.017, 0.106)	(0.014, 0.047)	(0.013, 0.034)
Photocopier development						
Sampling case	10	12	13	11	9	6
GM (mg/m^3^)	0.018	0.016	0.014	0.014	0.014	0.016
(GSD)	(1.303)	(1.107)	(2.210)	(1.326)	(1.432)	(1.038)
Median (mg/m^3^)	0.017	0.016	0.011	0.015	0.015	0.016
(quartile 1, quartile 3)	(0.016, 0.020)	(0.014, 0.018)	(0.009, 0.017)	(0.011, 0.016)	(0.010, 0.019)	(0.015, 0.016)
Toner and photocopier recycling						
Sampling case	23	29	33	34	50	44
GM (mg/m^3^)	0.569	0.236	0.075	0.066	0.067	0.029
(GSD)	(3.704)	(2.562)	(3.169)	(2.879)	(2.908)	(2.638)
Median (mg/m^3^)	1.054	0.211	0.060	0.051	0.065	0.021
(quartile 1, quartile 3)	(0.1 10, 1.420)	(0.139, 0.363)	(0.036, 0.137)	(0.037, 0.151)	(0.030, 0.126)	(0.014, 0.050)
Customer service						
Sampling case	18	22	23	18	16	19
GM (mg/m^3^)	0.020	0.018	0.013	0.013	0.013	0.013
(GSD)	(1.308)	(1.635)	(1.370)	(1.419)	(1.391)	(1.471)
Median (mg/m^3^)	0.019	0.016	0.013	0.015	0.014	0.014
(quartile 1, quartile 3)	(0.016, 0.022)	(0.015, 0.019)	(0.010, 0.016)	(0.009, 0.016)	(0.009, 0.017)	(0.009, 0.016)

^#^Sampling case: about 5 workers in one workplace were randomly selected and conducted personal exposure measurement.

For toner manufacturing process and toner and photocopier recycling process, the workplaces were subdivided and the sampling cases increased.

On the other hand, toner development process, photocopier development process, and customer service process had a few work units.

^##^GM: geometric mean. GSD: geomrtric standard deviation.

**Table 2:** Demographics of subjects at the baseline.

	All subjects	Toner-handling group	Never-toner-handling group	*P* value^#^
Subjects number	1504	1071	415	
Age				
mean (SD^##^)	38.6 (6.90)	39.0 (7.08)	37.8 (6.29)	<0.01**
Smoking status				
Current smoker (%)	731 (49.3%)	541 (50.7%)	190 (45.8%)	
Exsmoker (%)	260 (17.5%)	185 (17.3%)	75 (18.1%)	0.22
Never smoker (%)	492 (21.4%)	342 (32.0%)	150 (32.0%)	
Morbidity				
Asthma (%)	146 (9.8%)	107 (10.0%)	39 (9.4%)	0.77
Allergic rhinitis^###^ (%)	743 (50.0%)	543 (50.7%)	200 (48.2%)	0.42
Allergic dermatitis^###^ (%)	318 (21.4%)	231 (21.6%)	87 (21.0%)	0.83
Work history				
Handling of organic compounds (%)	254 (17.2%)	240 (22.6%)	14 (3.4%)	<0.01**

^#^ *P* value of Student's *t*-test and chi-square test, *P < 0.05 and **P < 0.01.

^##^Standard deviation.

^###^Allergic rhinitis includes pollinosis, and allergic dermatitis includes atopic dermatitis.

18 subjects were excluded from the analysis due to lack of the work history and 1486 subjects were analyzed.

Among 1486 subjects, smoking status of 3 subjects was missed.

Toner-handling workers who engaged in customer service used an alcoholic organic solvent to clean photocopier machines. Toner-handling workers who engaged in toner manufacturing responded that they used organic compounds during toner producing process, but organic compounds were used in the sealed production line and workers were not exposed in steady work.

**Table 3:** Prevalence of self-reported respiratory symptoms.

	1st survey			2nd survey			3rd survey			4th survey			5th survey			6th survey		
	Toner-handling group	Never-toner-handling group	*P* value^#^	Toner-handling group	Never-toner-handling group	*P* value^#^	Toner-handling group	Never-toner-handling group	*P*-value^#^	Toner-handling group	Never-toner-handling group	*P*-value^#^	Toner-handling group	Never-toner-handling group	*P*-value^#^	Toner-handling group	Never-toner-handling group	*P*-value^#^
Subjects number	1071	415		1045	388		1028	371		1016	361		993	352		986	342	
Persistent cough (%)	47 (4.4%)	19 (4.6%)	0.89	38 (3.6%)	17 (4.4%)	0.54	44 (4.3%)	14 (3.8%)	0.76	34 (3.3%)	18 (5.0%)	0.20	44 (4.4%)	9 (2.6%)	0.15	40 (4.1%)	12 (3.5%)	0.75
Persistent phlegm (%)	50 (4.7%)	12 (2.9%)	0.15	41 (3.9%)	13 (3.4%)	0.75	42 (4.1%)	8 (2.2%)	0.10	38 (3.7%)	10 (2.8%)	0.50	38 (3.8%)	7 (2.0%)	0.12	44 (4.5%)	10 (2.9%)	0.27
Persistent cough and phlegm (%)	18 (1.7%)	4 (1.0%)	0.47	7 (0.7%)	8 (2.1%)	0.04	16 (1.6%)	6 (1.6%)	1.00	15 (1.5%)	7 (1.9%)	0.62	19 (1.9%)	2 (0.6%)	0.13	21 (2.1%)	4 (1.2%)	0.36
Wheezing without asthmatic response (%)	68 (6.3%)	18 (4.3%)	0.17	57 (5.5%)	10 (2.6%)	0.02*	45 (4.4%)	10 (2.7%)	0.16	48 (4.7%)	10 (2.8%)	0.13	29 (2.9%)	10 (2.8%)	1.00	24 (2.4%)	13 (3.8%)	0.19
Breathlessness (%)	111 (10.4%)	22 (5.3%)	<0.01**	92 (8.8%)	19 (4.9%)	0.01*	80 (7.8%)	12 (3.2%)	<0.01**	69 (6.8%)	16 (4.4%)	0.13	66 (6.6%)	11 (3.1%)	0.02*	57 (5.8%)	8 (2.3%)	<0.01**

^#^ *P* value of Chi-square test, *P < 0.05 and **P < 0.01.

**Table 4:** The results of GEE on self-reported respiratory symptom (toner exposure, smoking status, organic compounds, allergic diseases, and age).

Dependent variable	Independent variable	Category	Odds ratio	95% confidence interval	
				Lower	Upper
Persistent cough	Toner exposure	Toner-handling group	0.95	0.61	1.47
		Never-toner-handling group	1.00		
	Smoking status	Current smoker	2.42*	1.55	3.79
		Ex-smoker	1.09	0.62	1.92
		Never smoker	1.00		
	Handling of organic compounds	+	1.08*	0.71	0.71
		−	1.00		
	Morbidity of asthma	+	2.46*	1.25	4.86
		−	1.00		
	Morbidity of allergic rhinitis	+	1.48*	1.07	2.05
		−	1.00		
	Morbidity of allergic dermatitis	+	1.56	0.93	2.62
		−	1.00		
	Age	1 year	1.02	0.99	1.05

Persistent phlegm	Toner exposure	Toner-handling group	1.31	0.86	2.01
		Never-toner-handling group	1.00		
	Smoking status	Current smoker	3.08*	1.93	4.91
		Ex-smoker	0.82	0.48	1.41
		Never smoker	1.00		
	Handling of organic compounds	+	1.02	0.75	1.40
		−	1.00		
	Morbidity of asthma	+	2.34*	1.39	3.95
		−	1.00		
	Morbidity of allergic rhinitis	+	1.45*	1.04	2.03
		−	1.00		
	Morbidity of allergic dermatitis	+	1.20	0.71	2.01
		−	1.00		
	Age	1 year	1.05*	1.02	1.08

Persistent cough and phlegm	Toner exposure	Toner-handling group	0.84	0.45	1.56
		Never-toner-handling group	1.00		
	Smoking status	Current smoker	3.31*	1.80	6.08
		Ex-smoker	0.61	0.26	1.43
		Never smoker	1.00		
	Handling of organic compounds	+	0.90	0.52	1.55
		−	1.00		
	Morbidity of asthma	+	3.19*	1.37	7.45
		−	1.00		
	Morbidity of allergic rhinitis	+	1.57	1.00	2.46
		−	1.00		
	Morbidity of allergic dermatitis	+	1.45	0.71	2.96
		−	1.00		
	Age	1 year	1.08	1.04	1.13

Wheezing without asthmatic response	Toner exposure	Toner-handling group	1.31	0.89	1.92
		Never-toner-handling group	1.00		
	Smoking status	Current smoker	2.73*	1.72	4.33
		Ex-smoker	1.17	0.67	2.07
		Never smoker	1.00		
	Handling of organic compounds	+	1.07	0.72	1.57
		−	1.00	
	Morbidity of asthma	+	8.26*	5.25	13.01
		−	1.00		
	Morbidity of allergic rhinitis	+	1.20	0.81	1.79
		−	1.00		
	Morbidity of allergic dermatitis	+	1.90*	1.07	3.38
		−	1.00		
	Age	1 year	1.05*	1.02	1.08

Breathlessness	Toner exposure	Toner-handling group	1.87*	1.29	2.71
		Never-toner-handling group	1.00		
	Smoking status	Current smoker	3.16*	2.19	4.56
		Ex-smoker	1.48	0.95	2.29
		Never smoker	1.00		
	Handling of organic compounds	+	1.28	0.95	1.72
		−	1.00		
	Morbidity of asthma	+	1.51	0.82	2.80
		−	1.00		
	Morbidity of allergic rhinitis	+	1.22	0.92	1.64
		−	1.00		
	Morbidity of allergic dermatitis	+	1.44	0.95	2.18
		−	1.00		
	Age	1 year	1.06*	1.04	1.08

*In case the independent variables had effects on the results.

**Table 5:** The results of GEE on self-reported respiratory symptom (toner exposure and pulmonary function indices).

Dependent variable	Independent variable	Category	Odds ratio	95% confidence interval	
				Lower	Upper
Persistent cough	Toner exposure	Toner-handling group	0.90	0.58	1.38
		Never-toner-handling group	1.00		
	%VC	1%	1.02	0.99	1.04
	%FEV_1.0_	1%	0.98	0.96	1.01

Persistent phlegm	Toner exposure	Toner-handling group	1.43	0.94	2.18
		Never-toner-handling group	1.00		
	%VC	1%	1.03	1.00	1.06
	%FEV_1.0_	1%	0.97	0.94	1.01

Persistent cough and phlegm	Toner exposure	Toner-handling group	1.15	0.60	2.19
		Never-toner-handling group	1.00		
	%VC	1%	1.04*	1.00	1.09
	%FEV_1.0_	1%	0.95*	0.91	1.00

Wheezing without asthmatic response	Toner exposure	Toner-handling group	1.44	0.97	2.15
		Never-toner-handling group	1.00		
	%VC	1%	1.08*	1.05	1.10
	%FEV_1.0_	1%	0.92*	0.90	0.94

Breathlessness	Toner exposure	Toner-handling group	1.98*	1.39	2.81
		Never-toner-handling group	1.00		
	%VC	1%	1.00	0.98	1.02
	%FEV_1.0_	1%	1.00	0.98	1.02

*In case the independent variables had effects on the results.

**Table 6:** Relationship between %FEV_1.0_ and breathlessness.

	1st survey	2nd survey	3rd survey	4th survey	5th survey	6th survey
Toner-handling group	24 (21.6%)	7 (8.1%)	11 (15.1%)	6 (9.1%)	9 (15.3%)	8 (14.8%)
Never-toner-handling group	2 (9.1%)	4 (25.0%)	0 (0.0%)	1 (7.7%)	2 (20.0%)	1 (14.3%)
*P* value^#^	0.24	0.07	0.34	1.00	0.66	1.00

^#^ *P* value of chi-square test; *P < 0.05 and **P < 0.01.

The percentage in the table was calculated by the following formula; the number of subjects with %FEV_1.0_ < 80% ∗ 100/the number of subjects with breathlessness.
